# Supplementary material for: Stratospheric impacts on dust transport and air pollution in West Africa and the Eastern Mediterranean
Source: Nat Commun. 2022 Dec 14;13:7744. doi: 10.1038/s41467-022-35403-1 (PMC9750965; doi:10.1038/s41467-022-35403-1)
Supplement: Supplementary file 1 — Supplementary Information [file 41467_2022_35403_MOESM1_ESM.pdf]

Supplementary Information for "Stratospheric impacts on dust transport and air pollution in West Africa and the Eastern Mediterranean"

Ying Dai[1], Peter Hitchcock[1*], Natalie M. Mahowald[1], Daniela I.V. Domeisen[2,3], Douglas S. Hamilton[1,4], Longlei Li[1], Beatrice Marticorena[5], Maria Kanakidou[6,7,8], Nikolaos Mihalopoulos[6,9], Adwoa Aboagye-Okyere[1]

[1]Department of Earth and Atmospheric Sciences, Cornell University, Ithaca, NY 14853, USA

[2]University of Lausanne, Lausanne, Switzerland

[3]ETH Zurich, Zurich, Switzerland

[4]Present address: Department of Marine, Earth, and Atmospheric Science, NC State University, Raleigh, NC, USA

[5]Laboratoire Interuniversitaire des Systèmes Atmosphériques, Universités Paris Est-Paris Diderot-Paris 7, UMR CNRS 7583, Créteil, France

[6]Environmental Chemical Processes Laboratory (ECPL), Department of Chemistry, University of Crete, Heraklion, Greece

[7]Center of Studies of Air quality and Climate Change, Institute for Chemical Engineering Sciences, Foundation for Research and Technology Hellas, Patras, Greece

[8]Excellence Chair, Institute of Environmental Physics, University of Bremen, Bremen, Germany

[9]Institute for Environmental Research and Sustainable Development, National Observatory of Athens, Pendeli, Greece

November 22, 2022, 8:52pm

Contents of this file

1. Supplementary Figures 1 to 8
2. Supplementary Tables 1 to 4
3. Supplementary Note 1

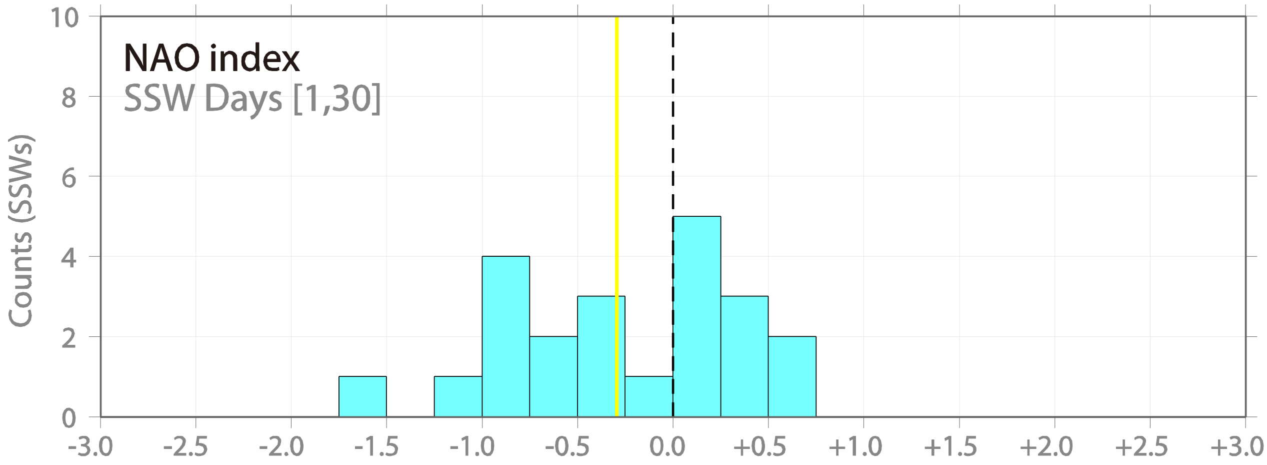

Supplementary Figure 1. Histogram of the North Atlantic Oscillation (NAO) index averaged during each of the 22 sudden stratospheric warming (SSW) episodes over the 1980/81-2013/14 extended winters (from November to March, NDJFM). The yellow vertical line indicates the average value of the index over all SSWs, which is slightly greater than -0.3. The NAO index is defined from the difference between normalized sea level pressure (SLP) between Lisbon, Portugal, and Stykkisholmur, Iceland. The SLP field comes from the MERRA2 reanalysis.

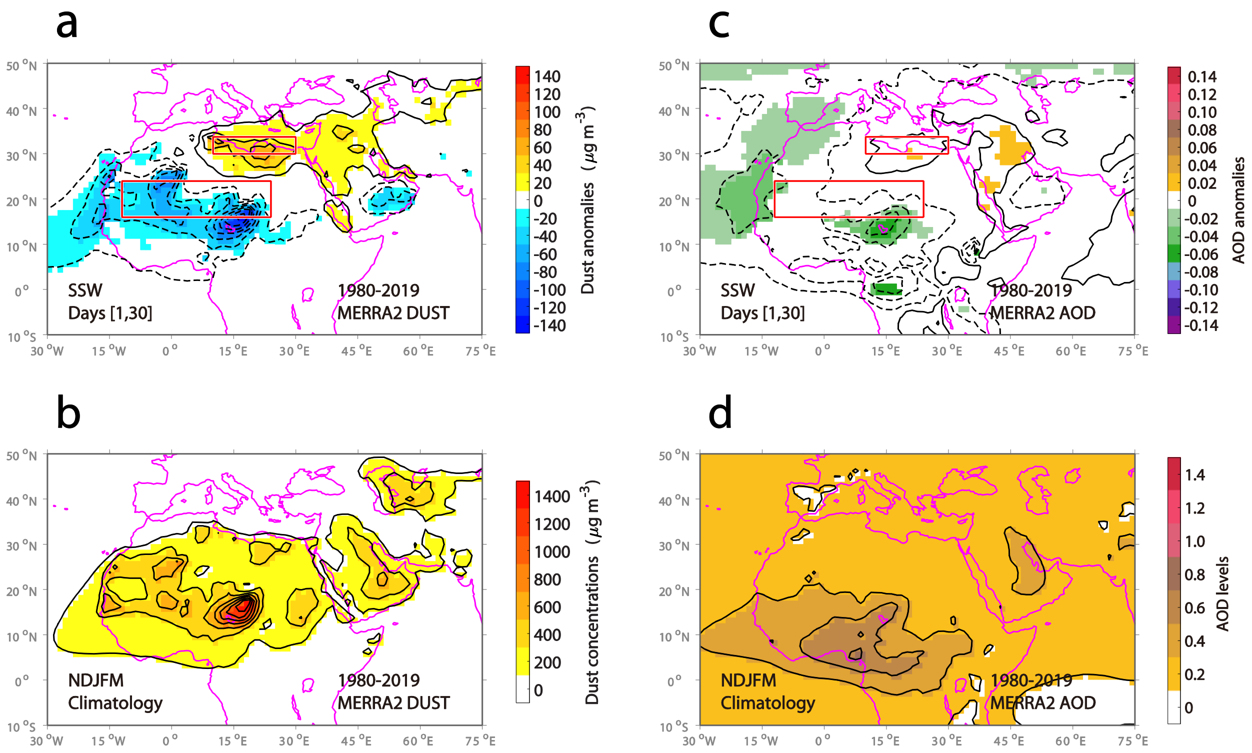

Supplementary Figure 2. (a) Composites of MERRA2 surface dust concentration anomalies (units: $\mu g\,m^{-3}$) during 24 Sudden Stratospheric Warming (SSW) episodes over the 1980/81-2019/20 extended winters (from November to March, NDJFM). Shadings indicate anomalies that are statistically significant at the $p < 0.10$ level based on a two-tailed Monte Carlo test. The two red boxes indicate the Eastern Mediterranean (30°-33.75°N, 10°-30°E) and West Africa (16.25°-23.75°N, 12.5°-23.75°E) regions, which are the same as the two red boxes in Figure 1c. (b) The corresponding extended wintertime (NDJFM) climatologies (units: $\mu g\,m^{-3}$). (c-d) The same as a-b but for MERRA2 aerosol optical depth (AOD). Made with Natural Earth. Free vector and raster map data @ naturalearthdata.com.

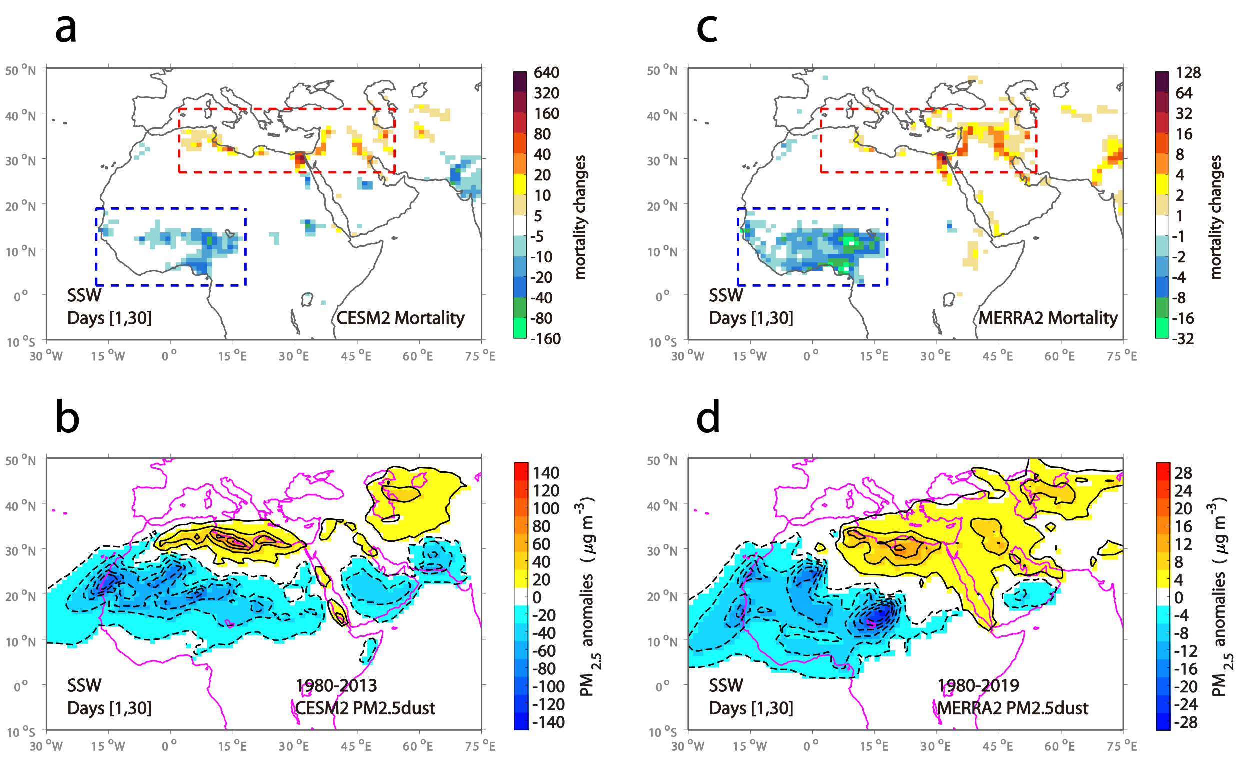

Supplementary Figure 3. (Upper) Changes in premature deaths [per sudden stratospheric warming (SSW) event] estimated from SSW-induced changes in dust-source fine particulate ($PM_{2.5}$) concentrations from (a) CESM2 and (c) MERRA2. Warm and cold shadings indicate positive and negative anomalies, respectively (note the non-linear color scale). The red rectangular box indicates the Mediterranean region (27°-41°N, 2°-54°E) with additional deaths, and the blue rectangular box indicates the West Africa region (2°-19°N, 18°W-18°E) with reduced deaths. The two boxes here, indicating the Mediterranean and West Africa regions with considerable changes in premature deaths, differ somewhat from those in Figure 1c and Supplementary Figure 2a which correspond to regions with large changes in dust concentrations. This is because, compared to changes in dust concentrations, the resulting changes in premature deaths tend to move towards large population centers. (Lower) SSW-induced changes in dust-source $PM_{2.5}$ concentrations (per SSW event) from (b) CESM2 and (d) MERRA2. Different colorbars are used for CESM2 and MERRA2. Made with Natural Earth. Free vector and raster map data @ naturalearthdata.com.

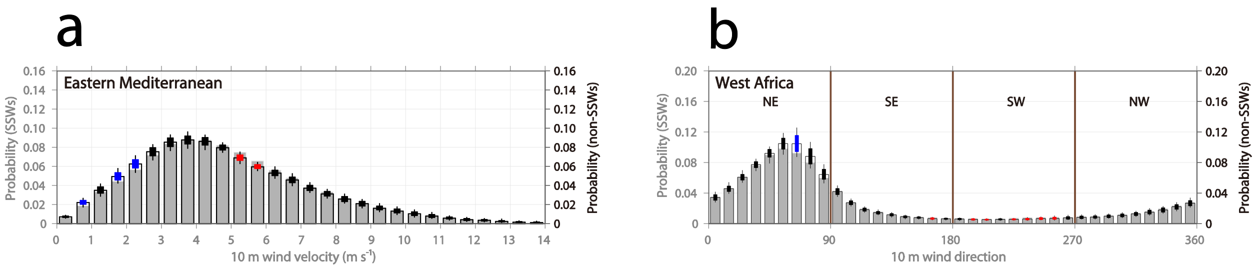

Supplementary Figure 4. The same as Figure 2 (b), (d) but for (a) surface wind velocities within the Eastern Mediterranean and (b) surface wind directions within West Africa. The Eastern Mediterranean and West Africa correspond to the two red boxes shown in Figure 1c. The surface wind fields come from the MERRA2 reanalysis.

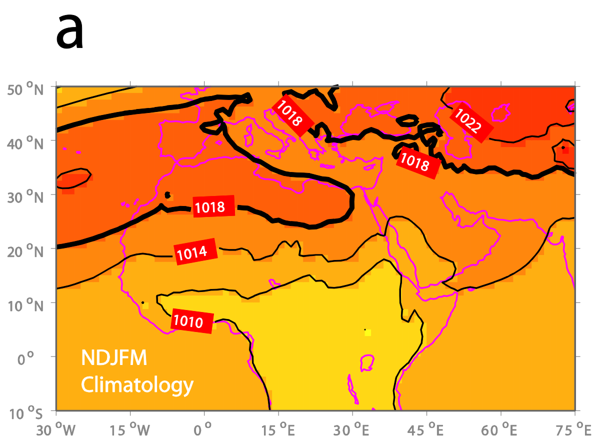

Supplementary Figure 5. (a) The extended wintertime (from November to March, NDJFM) climatology of the full sea level pressure (SLP) field. (b) The full SLP field during the sudden stratospheric warming (SSW) episodes. The contour lines in panel a are the same as those in Figure 1b. The lines in bold indicate the 1018 hPa contour lines, which correspond to a belt of high pressure around 30°N and sits at the divergence of trade winds and the westerlies (see wind arrows in Figure 1b). These features indicate that the 1018 hPa contour line matches well with the descending branch of the Hadley cell because the trade winds are the return flow of the Hadley cell under the influence of the Coriolis force. We therefore plot the subtropical ridge in the schematic diagram (red contour lines in Figure 3) following the 1018 hPa contour lines shown here, given the fact that the subtropical ridge is a high-pressure area caused by the descending branch of the Hadley cell. Made with Natural Earth. Free vector and raster map data @ naturalearthdata.com.

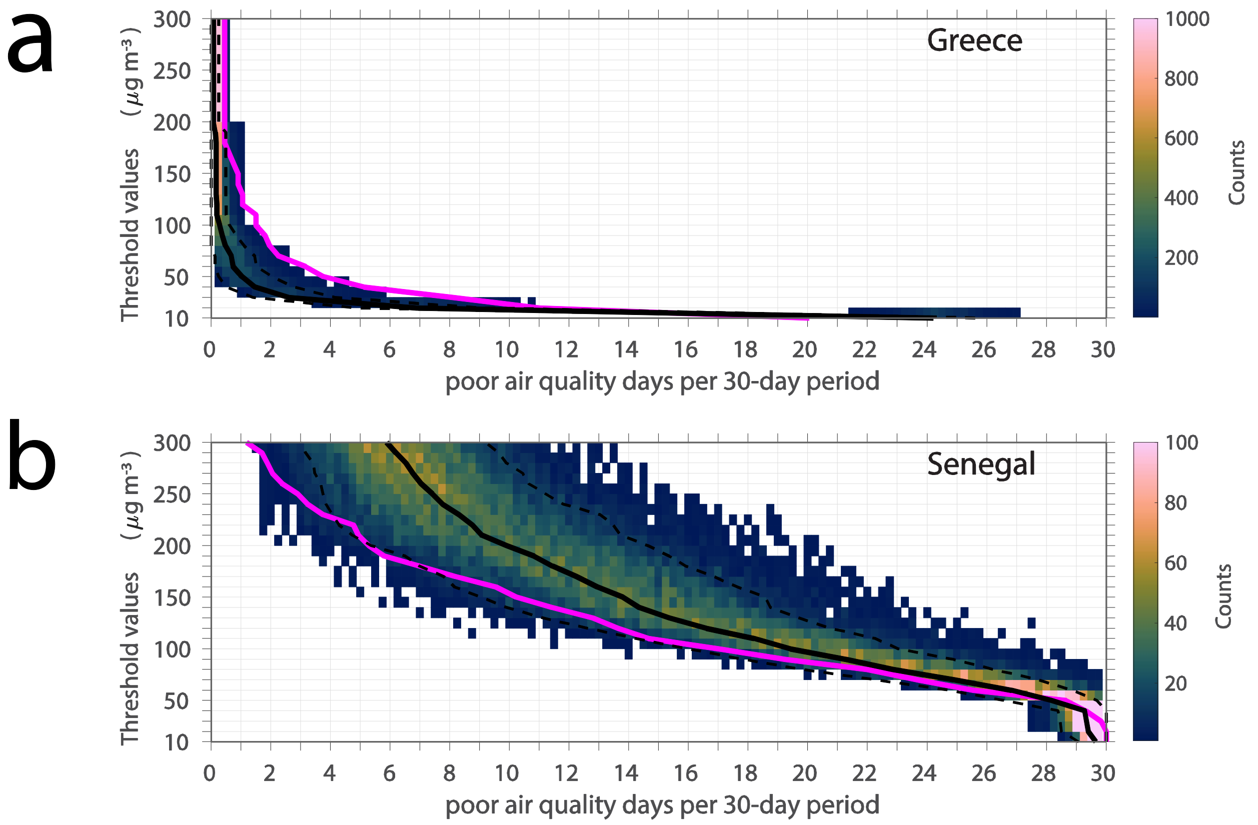

Supplementary Figure 6. The expected number of poor air quality days per 30-day period in (a) Finokalia, Greece and (b) M'Bour, Senegal. At a given threshold value, poor air quality days are defined when the daily mean inhalable particulate (PM_{10}) concentrations exceed this standard. The standards used range from 10 $\mu g\, m^{-3}$ to 300 $\mu g\, m^{-3}$ with an increment of 10 $\mu g\, m^{-3}$ (see the y-axis). The thick pink curve indicates the expected number of poor air quality days per 30-day period during sudden stratospheric warming (SSW) episodes. For each threshold value, the shadings show the spread of the number of poor air quality days per 30-day period during 1000 sets of non-SSW episodes, with the thick black curve indicating the non-SSW mean and the dashed black curves indicating the 5th-95th percentile confidence interval derived from the non-SSW spread.

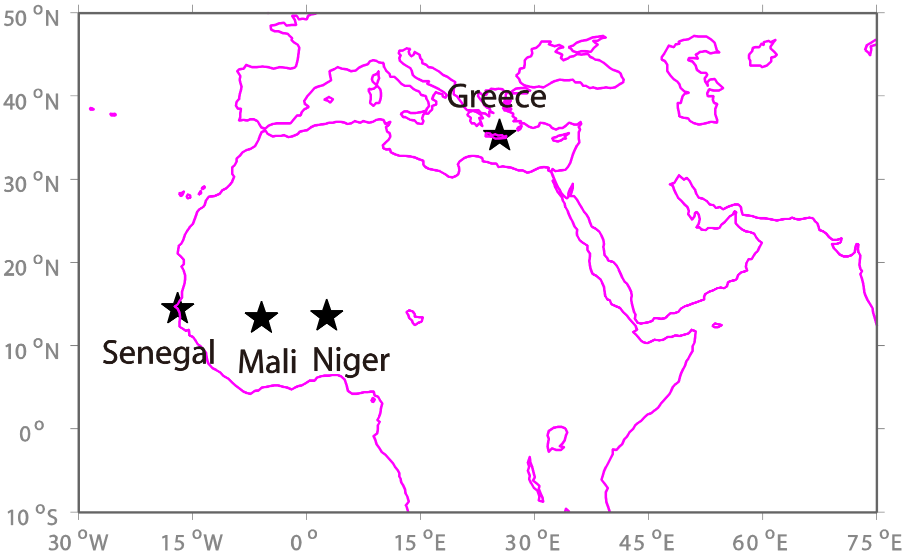

Supplementary Figure 7. Location of the four observational stations. These stations are situated at Greece (35°20′N, 25°40′E), Senegal (14°42′N, 16°28′W), Mali (13°17′N, 5°56′W), and Niger (13°31′N, 2°38′E), respectively. Made with Natural Earth. Free vector and raster map data @ naturalearthdata.com.

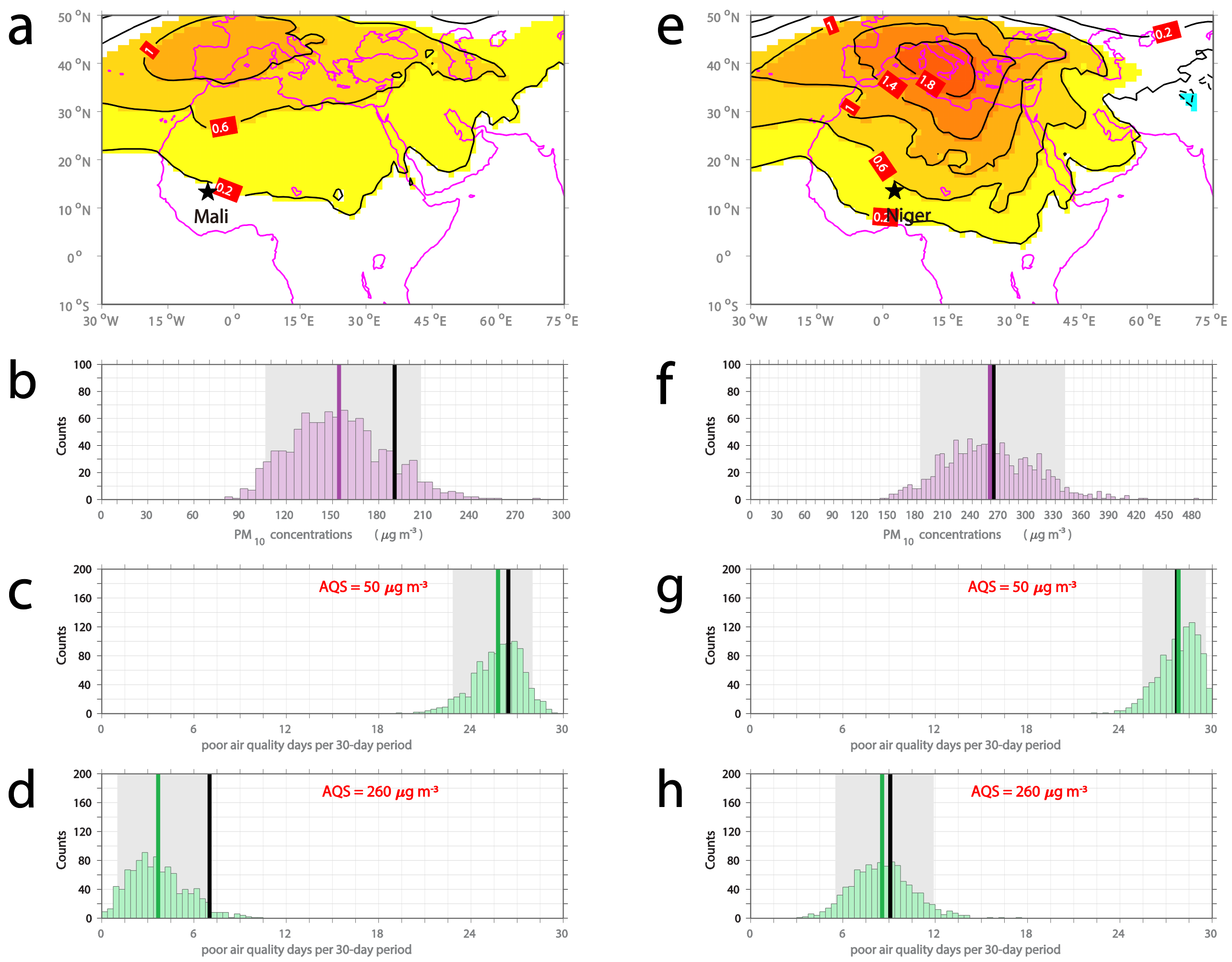

Supplementary Figure 8. The same as Figure 4 but for the observational daily time-series of surface inhalable particulate (PM_{10}) concentrations collected in (a-d) Cinzana, Mali and (e-h) Banizoumbou, Niger. Made with Natural Earth. Free vector and raster map data @ naturalearthdata.com.

Supplementary Table 1. Sudden stratospheric warming-caused (SSW-caused) changes relative to the non-SSW mean in the level of surface dust concentrations and in the number of high dust pollution days on which the specified daily thresholds 250, 500, and 1000 $\mu g\,m^{-3}$ are exceeded. The Eastern Mediterranean and West Africa correspond to the two red boxes shown in Figure 1c and Supplementary Figure 2a. The changes obtained from CESM2 and MERRA2 are shown respectively. A combined range of these changes is also shown to provide an estimate of the model uncertainty, which is obtained by taking the highest and lowest changes of the two models and then rounding these changes to the nearest ten. For each model, the subscript and superscript numbers denote the sampling uncertainty ranges taken as the 5th–95th percentile confidence interval. This involves randomly resampling, with replacement, 22 SSWs from the 22 available in CESM2 (or 24 SSWs from the 24 available in MERRA2), and recomputing the composite-mean SSW-caused changes 1000 times. The bottom row illustrates the extended wintertime climatologies (all days from November to March) of surface dust concentrations in the Eastern Mediterranean and West Africa derived from CESM2 and MERRA2.

	Eastern Mediterranean			West Africa		
	CESM2	MERRA2	combined range	CESM2	MERRA2	combined range
Surface dust concentrations	$+19_{6}^{33}$%	$+28_{16}^{39}$%	[20%,30%]	-16_{-21}^{-12}%	-12_{-16}^{-7}%	[-20%,-10%]
High pollution days (⩾250)	$+20_{10}^{29}$%	$+37_{23}^{51}$%		-10_{-14}^{-5}%	-9_{-15}^{-3}%	
High pollution days (⩾500)	$+26_{13}^{39}$%	$+41_{20}^{64}$%	[20%,40%]	-19_{-25}^{-14}%	-24_{-31}^{-16}%	[-30%,-10%]
High pollution days (⩾1000)	$+27_{9}^{47}$%	$+29_{1}^{59}$%		-32_{-40}^{-23}%	-31_{-41}^{-21}%	
Climatology ($\mu g\ m^{-3}$)	372	159		573	381	

Supplementary Table 2. Sudden stratospheric warming-caused (SSW-caused) changes relative to the non-SSW mean in the level of aerosol optical depth (AOD) from AERONET and MERRA2 at the four observational sites. For MERRA2 AOD, two columns are displayed, with the first and second column showing changes derived from the time-series at the AERONET length and at the FULL length, respectively. The AERONET length for each site is listed in Supplementary Table 3 and the FULL length of MERRA2 covers years 1980-2020 with 24 SSWs in total. When the level of AOD during SSW episodes falls outside the 5th–95th percentile confidence interval derived from the non-SSW spread, the SSW-caused changes are marked with an asterisk.

Site	AERONET	MERRA2 at AERONET length	MERRA2 at FULL length
Greece	+10%	+13%	0%
Senegal	-11%	-9%	-12%*
Mali	+8%	+2%	-8%
Niger	-11%	-10%*	-10%*

Supplementary Table 3. Time periods and number of sudden stratospheric warming events (SSWs) covered by station observations. In the table, PM_{10} refers to inhalable particulate, and AOD refers to aerosol optical depth.

Site	PM_{10}		AERONET AOD	
	Temporal coverage	# of SSWs	Temporal coverage	# of SSWs
Greece	2004-2019	9	2003-2017	8
Senegal	2006-2018	8	2000-2020	14
Mali	2006-2018	8	2004-2020	9
Niger	2006-2017	7	1995-2020	17

Supplementary Table 4. The central dates of Northern Hemisphere sudden stratospheric warming events (SSWs) detected in the MERRA2 reanalysis.

#	central dates of SSWs
1	4 Dec 1981
2	24 Feb 1984
3	1 Jan 1985
4	23 Jan 1987
5	8 Dec 1987
6	14 Mar 1988
7	21 Feb 1989
8	15 Dec 1998
9	26 Feb 1999
10	20 Mar 2000
11	11 Feb 2001
12	30 Dec 2001
13	17 Feb 2002
14	18 Jan 2003
15	5 Jan 2004
16	21 Jan 2006
17	24 Feb 2007
18	22 Feb 2008
19	24 Jan 2009
20	9 Feb 2010
21	24 Mar 2010
22	6 Jan 2013
23	12 Feb 2018
24	2 Jan 2019

Supplementary Note 1: Uncertainty in observed air quality responses

The observed air quality responses at the background stations differ in terms of the magnitude and sometimes even the sign, depending on the source of air quality related field, the time period used, and the location of the station.

In Senegal, using PM_{10} concentrations and AOD (AERONET and MERRA2) yields consistent responses. All these air quality related fields suggest better air quality in Senegal during SSW episodes, as can be seen from the decline in air quality indicators (see Figure 4f,h and Supplementary Figure 6b) and the decrease in AOD (reduced by roughly 10%, see Supplementary Table 2). The observed improvement in air quality in Senegal is consistent with the simulated reduction in dust burden in West Africa (Figure 1c).

In Mali, PM_{10} concentrations and AERONET AOD suggest somewhat worsened air quality during SSW episodes, as indicated by the rise in air quality indicators (Supplementary Figure 8b-d) and the increase in AERONET AOD (Supplementary Table 2). By contrast, MERRA2 AOD (at full length) suggest better air quality during SSW episodes, as indicated by the decrease in MERRA2 AOD (Supplementary Table 2). Given the fact that MERRA2 AOD (at full length) cover 24 SSW events, there is reason to believe the better air quality suggested by MERRA2 AOD (at full length) is more likely than the worse air quality suggested by PM_{10} concentrations and AERONET AOD, which cover only 8 and 9 SSW events, respectively (Supplementary Table 3).

In Niger, while the PM_{10} concentrations show a neutral air quality response to SSWs (both air quality indicators lie close to the non-SSW mean, see Supplementary Figure 8f-h), the AERONET and MERRA2 AOD consistently suggest better air quality during SSW episodes, as can be seen from the roughly 10% decrease in AOD (Supplementary Table 2). Again, it is reasonable to

believe the better air quality suggested by AOD is more likely than the neutral response suggested by PM_{10} concentrations, since the AERONET and MERRA2 AOD cover 17 and 24 SSW events, respectively, while the PM_{10} concentrations cover only 7 SSW events (Supplementary Table 3).

In Greece, PM_{10} concentrations, AERONET AOD, and MERRA2 AOD (at AERONET length) consistently suggest worsened air quality during SSW episodes, as indicated by the rise in air quality indicators (see Figure 4b-d and Supplementary Figure 6a) and the increase in AOD (increased by roughly 10-15%, see Supplementary Table 2). However, MERRA2 AOD (at full length) suggests neutral air quality response to SSWs (Supplementary Table 2) at this observational site, due to the fact that the single site in Greece falls outside the region with enhanced AOD signal in the Eastern Mediterranean (Supplementary Figure 2c). Note that MERRA2 AOD (at full length) shows a dipolar structure response to SSWs with enhanced AOD signal in the Eastern Mediterranean and reduced AOD signal in West Africa (Supplementary Figure 2c), consistent with the air quality responses suggested by other observed and simulated air quality related fields.

Overall, the observational evidence indicates better air quality in West Africa and worse air quality in the Eastern Mediterranean during SSW episodes, consistent with the dipolar dust response derived from the modeled datasets. As to the uncertainties in observed air quality responses, they largely arise from the potential impacts of sampling error from short observational records and inadequate representativeness of limited background stations, and are likely to be reduced when longer-term records collected at more extensive sites become available.
